# Transgenic Improvement for Biotic Resistance of Crops

**DOI:** 10.3390/ijms232214370

**Published:** 2022-11-19

**Authors:** Haoqiang Yu, Yingge Wang, Fengling Fu, Wanchen Li

**Affiliations:** Maize Research Institute, Sichuan Agricultural University, Ya’an 611130, China

**Keywords:** biotic, crop, improvement, transgene

## Abstract

Biotic constraints, including pathogenic fungi, viruses and bacteria, herbivory insects, as well as parasitic nematodes, cause significant yield loss and quality deterioration of crops. The effect of conventional management of these biotic constraints is limited. The advances in transgenic technologies provide a direct and directional approach to improve crops for biotic resistance. More than a hundred transgenic events and hundreds of cultivars resistant to herbivory insects, pathogenic viruses, and fungi have been developed by the heterologous expression of exogenous genes and RNAi, authorized for cultivation and market, and resulted in a significant reduction in yield loss and quality deterioration. However, the exploration of transgenic improvement for resistance to bacteria and nematodes by overexpression of endogenous genes and RNAi remains at the testing stage. Recent advances in RNAi and CRISPR/Cas technologies open up possibilities to improve the resistance of crops to pathogenic bacteria and plant parasitic nematodes, as well as other biotic constraints.

## 1. Transgenic Improvement Is Effective to Control Biotic Constraints

### 1.1. Biotic Constraints to Crops

**Figure 1:**
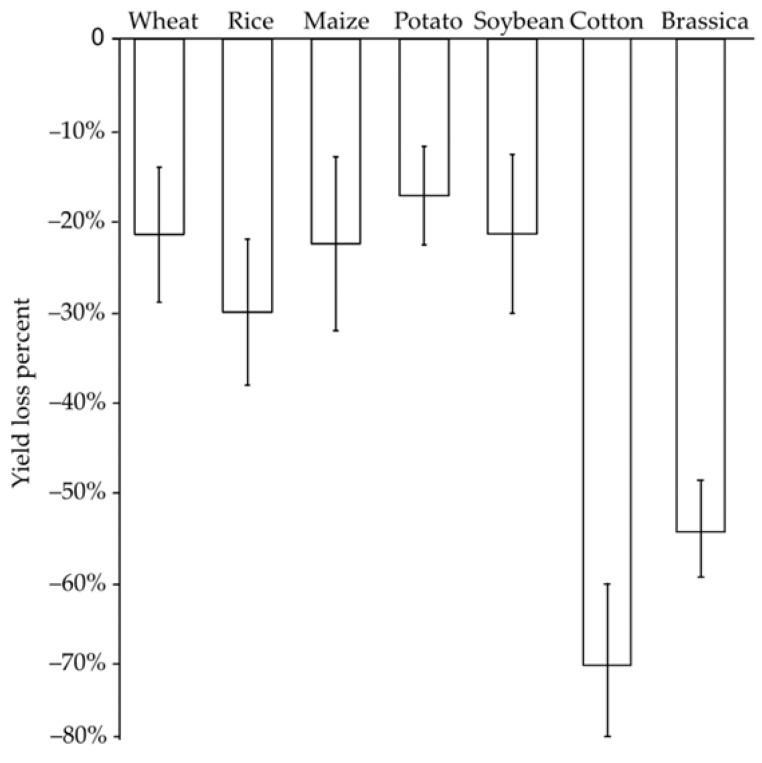
Global yield loss to pathogens and pests on major food crops.

As sessile organisms, crop plants have to cope with other living organisms in their environment. Except for a few, such as rhizobia, which are beneficial to their hosts, most of the others cause some damage to crop plants such as pathogenic fungi, viruses, bacteria, herbivory insects, as well as parasitic nematodes. They are classified as biotic constraints to crop plants [1,2,3,4,5]. Pathogenic fungi and bacteria are mostly intracellular pathogens. They invade different organs of the plant during the interaction with the host, live inside and kill the host cells using toxic enzymes or compounds such as necrotrophs or initially feed on plants parasitically as biotrophs or hemi-biotrophs, leading to vascular wilts, leaf spots, and cankers, among other symptoms [4,5]. Pathogenic viruses produce not only local lesions but also systemic damage that causes stunting, chlorosis, and malformations, although they rarely kill their hosts. They have a very efficient system of dissemination through vector transmission by insects, arthropods, and nematodes [6]. Herbivory insects, as well as mites, damage plants through feeding or egg-laying. Piercing–sucking insects can also act as virus vectors, transmitting them to plants through their styles [1,7]. Plant-parasitic nematodes attack the root system, withdraw the nutrients of plant cells, and alter plant development, physiology, and immunity, resulting in leaf necrosis and chlorosis, plant wilting, and stunting, and enhanced susceptibility to other pathogens [8,9,10]. Several economically important genera parasitize various crop plants. The root-knot, root lesion, and cyst nematodes of *Heteroderidae* are the three most economically damaging genera of plant-parasitic nematodes and lead to significant yield loss [10]. In order to deal with these biotic stresses, plants have evolved an advanced immune system, such as physical barriers (waxes, thick cuticles, and specialized trichomes) to prevent pathogen settling and herbivory feeding, chemical compounds to protect against herbivory and pathogen infection, and immune responses that activate plant defense mechanisms in a much more effective way [11,12,13,14,15]. However, these mechanisms cannot completely resist pathogen and herbivory damage, which still affect crop development and lead to a yield loss of 17–30% (Figure 1) and significant quality deterioration, and are irrefutably the most serious threat to worldwide food security [1,7,16,17,18,19]. In the *Brassica* crops, which can be invaded and fed by numerous pathogens and herbivory insects, the yield loss is estimated as high as 50–60% [20,21,22].

### 1.2. Conventional Management

To meet the food demand of the booming world population, sustainable agriculture makes great efforts to reduce the yield loss and quality deterioration of crops without compromising the biotic constraints [1,23]. In agricultural practice, the conventional approaches are traditional breeding for resistance and application of agrochemicals (fungicides, bactericides, nematicides, and insecticides), assisted by comprehensive field management and biological control [24]. The application of resistant cultivars with broad-spectrum and durable resistance is the most cost-effective, environmentally friendly, and less labor-intensive [25,26,27,28]. However, traditional breeding heavily depends on germplasm resources. It is effective for the improvement of vertical resistance to specific parasitic pathotypes of biotrophic pathogens, such as rust fungi *Puccinia striiformis*, *P*. *triticina*, and *P*. *graminis* (pathogens of stripe, leaf, and stem rust of wheat, respectively) and *Phytophthora infestans* (pathogen of potato late-blight) [29,30,31,32]. This kind of resistance is complete but can be easily evaded due to the continuous accumulating mutations of pathogens, leading to the breakdown of resistant varieties after their commercial utilization [33,34,35,36]. For improvement of horizontal resistance to non-specific parasitic pathotypes of saprophytic pathogens, such as *Bhizoctonia solani* and *Hyaloperonospora parasitica* (pathogens of maize sheath blight and rape downy mildew, respectively), as well as pathogenic viruses and phytophagous insects, it is a great challenge to pyramid the multiple dominant resistant genes into a single cultivar for developing broad-spectrum and durable resistance [37,38,39,40,41,42]. This challenge occurs even with the advances in distant hybridization, artificial mutation, double haploid technology, somatic hybrid, and DNA marker-assisted genotyping and selection [38,39,43,44,45,46,47,48,49], due to the limitation of germplasm resources, the genetic background of minor polygenes, and negative correlation with yield and quality characteristics [50,51,52,53]. The application of agrochemicals is effective for the control of herbivory insects, but increases production costs and causes environmental pollution [54,55,56,57]. For the control of pathogenic fungi and bacteria, especially plant parasitic nematodes, conventional management is much more difficult. Comprehensive field management procedures, such as field sanitation, soil sterilization, crop rotation and fallowing, rouging, and the manual removal of infected plants, are only applied as assistive approaches, because of their labor input and limits in disease and insect eradication [16,58,59]. Biological control may not be compatible with the crop production system or the regulatory environment and remains at the testing stage, except for a few successful stories [60,61].

### 1.3. Transgenic Improvement

**Figure 2:**
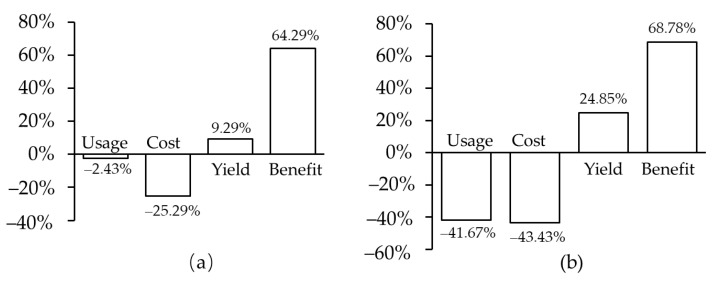
Benefits of herbicide-resistant (**a**) and insecticidal (**b**) transgenic crops.

Transgenic technology overcomes the hybridization barriers, utilizes a broader and more diverse range of desirable genes from genetically distant species, and provides a direct and directional approach to improve crops for biotic resistance in a careful target manner with minimal effect on beneficial soil microbes and environment [62,63,64]. It is praised as a second Green Revolution, hopeful for improving biotic resistance and other agronomical characteristics of crops and making a great contribution to food security [65,66]. Transgenic cultivars of crops are generated by the cloning of desirable genes, construction of expression vectors, genetic transformation of recipient cultivars, and identification of transgenic lines, so as to improve the original undesirable traits or endow them with new beneficial traits [65,66,67]. Transgenic technology is also used to modify or knock out some suboptimal genes to change their undesirable phenotypes [68,69,70,71]. After a strict assessment for food and environmental safety, transgenic cultivars with significant improvement in biotic resistance and other agronomical characteristics are authorized for cultivation and market [72]. According to the survey carried out by the International Service for the Acquisition of Agri-Biotech Applications (ISAAA, http://www.isaaa.org/gmapprovaldatabase/default.asp, accessed on 22 September 2022), 292 transgenic events and hundreds of varieties and hybrids between them of 32 cereals, vegetables, fruits, fibers, oils, trees, and forage crops have been authorized for cultivation and market, cultivated for 176.85 million hectares in more than thirty countries, and providing great profitability by increasing yield and reducing input in pesticides, labor, and machinery. They are harmless to humans, environmentally friendly, and favored by farmers worldwide [62,73,74]. A meta-analysis shows that the application of herbicide-resistant and insecticidal transgenic crops reduces the use of synthetic herbicide and pesticides by 2.43% and 41.67%, and their cost by 25.29% and 43.43%, increases the yield of crops by 9.29% and 24.85%, and benefits farmers by 64.29% and 68.78%, respectively (Figure 2) [75].

## 2. Commercial Release for Transgenic Resistance to Herbivory Insects, and Pathogenic Viruses, and Fungi

### 2.1. Resistance to Herbivory Insects

One of the most successful transgenic improvements for biotic resistance is the wide application of transgenic events resistant to herbivory insects, which used to be the major biotic constraint that caused a serious reduction in crop productivity and posed a great threat to food security in the world [7]. According to the survey carried out by ISAAA (http://www.isaaa.org/gmapprovaldatabase/default.asp, accessed on 22 September 2022), 93 single transgenic events (not included hybrids between them. The same below.) resistant to herbivory insects of lepidopteran, coleopteran, as well as hemipteran orders, have been authorized for cultivation and market. From them, hundreds of cotton, cowpea, eggplant, maize, potato, rice, soybean, sugarcane, and tomato varieties and hybrids have been developed and authorized for cultivation and market. Their application has reduced pesticide usage and increased yield and the benefits of crop production [73,74,75,76,77,78,79]. Ninety of these ninety-three transgenic events were transformed by the heterologous expression of one or more (for pyramiding resistance) of the insecticidal genes *Cry* (δ–endotoxin) from different strains of soil bacterium *Bacillus thuringiensis* [80], and one was transformed by the heterologous expression of the vegetative insecticidal protein gene *vip3* also from *B*. *thuringiensis*. Ten were simultaneously transformed by orthologs of the *Cry* gene from the other strains of *B*. *thuringiensis* or for pyramiding resistance. Three were simultaneously transformed by vegetative insecticidal protein genes *vip3*, *CpTI*, and *API*, as well as double-stranded RNA transcript of gene *Snf7* from Western corn rootworm (*Diabrotica virgifera*), respectively, for pyramiding resistance (Table 1) [81,82,83,84].

### 2.2. Resistance to Pathogenic Viruses

In the early years, the transgenic improvement for resistance to pathogenic viruses was focused on pathogen-derived resistance (PDR), whereby a sequence or part of the viral genome was introduced into the host plant [85,86,87,88,89]. After its discovery in the model non-parasitic nematode *Caenorhabditis elegans* and demonstration in transgenic improvement of vial resistance [90,91], RNA interference (RNAi) technology has rapidly emerged as the most effective strategy of transgenic improvement for resistance to plant viruses [91]. Synthetic DNA sequences expressing double-stranded RNAs (dsRNAs) for interference of the housekeeping genes of pathogenic viruses, such as the genes of the coat protein (CP) [92,93,94], the replication protein (Rep) [95,96,97], as well as the movement protein (MP) [98,99]. According to the survey carried out by ISAAA (http://www.isaaa.org/gmapprovaldatabase/default.asp, accessed on 22 September 2022), twenty-five transgenic events of bean, papaya, plum, potato, squash, sweet pepper, and tomato resistant to pathogenic viruses were generated and authorized for cultivation and market (Table 2). All of them were transformed by double-stranded sequences transcribing the coat protein, replicase, or helicase of the pathogenic viruses themselves for RNAi [94,100,101,102]. In recent years, clustered regularly interspaced short palindromic repeats-cas (CRISPR/Cas) technology and its new advances attracted much attention in transgenic improvement for rival resistance [103,104,105,106]. Transgenic potato lines with broad-spectrum resistance were developed by introduction of a multiple gRNA cassette of CRSIPR/Cas13 system to target six genes of three potato virus strains [106].

### 2.3. Resistance to Pathogenic Fungi

A lot of attempts have been made to battle against a wide range of pathogenic fungi by transgenic improvement [107,108,109,110,111,112,113,114,115]. However, most these attempts were generated by the overexpression of endogenous genes resistant to pathogenic fungi [109,116,117]. Unfortunately, their resistance improvement was usually non-significant, highly specific against a few pathogens, and even showed reduced yield or growth vigor, due to the complex mechanisms and signaling networks of disease resistance [107,109,118]. Almost all of them remain at testing stage and few commercial applications have yet been achieved [107,119]. Much effort has been also paid to the heterologous expression of exogenous resistant genes. The immune receptor gene *AtRLP23* was introduced into potato and resulted in smaller lesions and reduced pathogen colonization when infected with *Phytophthora infestans* or *Sclerotinia sclerotiorum* [120]. The heterologous expression of the immune receptor genes *SlVe1* from tomato and *SmELR* from wild potato also enhanced resistance to *Verticillium* wilt in tobacco and cotton, and *Phytophthora infestans* in potato, respectively [121,122]. According to the survey carried out by ISAAA (http://www.isaaa.org/gmapprovaldatabase/default.asp, accessed on 22 September 2022), only four transgenic events for the resistance improvement for pathogenic fungi have been authorized for cultivation and market for their significant improvement of resistance to the potato late-blight pathogen (*Phytophthora infestans*). One (event SP951) was transformed by the heterologous expression of exogenous resistant genes *RB* from distant species *Solanum bulbocastanum* of the nightshade family. The resistance of the other three (W8, X17, and Y9) were conferred by the heterologous expression of exogenous gene *Rpi-vnt* from distant species *Solanum venturi* of the nightshade family, and pyramided by RNAi of endogenous genes *Asn1*, *Ppo5*, *Phl*, *R1*, and *Vinv* related to pathogenesis [123]. In recent times, a more efficient strategy was developed to combine multiple resistance genes in an expression cassette. These combined genes not only increased the resistance spectrum to fungal rust pathogen in the transgenic wheat lines, but also inherited a single genetic locus without segregation [124].

## 3. Exploration for Transgenic Resistance to Bacteria and Nematodes

### 3.1. Resistance to Pathogenic Bacteria

A lot of efforts have also been devoted to the creation of transgenic resistance to bacterial diseases [107,125,126]. In fact, antibacterial proteins, widely present in animals, plants, and bacteriophages, have a bactericidal action on a large range of Gram-negative and -positive bacteria. Their encoding genes have been cloned from genetic distant species and heterogeneously expressed in crops in attempts to confer resistance to bacterial diseases [125]. For example, the genes of cecropins (lytic peptides) were cloned from the giant silk moth and expressed in potato and tobacco plants. The transgenic lines showed delayed symptoms and reduced mortality following inoculation with *Ralstonia solanacearum* and *Pseudomonas solanacearum*, and *Pseudomonas syringae* (pathogen of bacterial wilt), respectively [127,128]. The heterogenous expression of the genes of lysozymes, lactoferrin, chitinase, the effectors of salicylic acid and jasmonic acid signaling, as well as other antibacterial peptide or phytoalexins genes, and silencing the expression of toxins, pectic enzymes, and exopolysaccharides genes of pathogenic bacteria have also been tried in the transgenic improvement of crops for bacterial resistance [129,130,131,132,133,134,135]. For example, the heterologous expression of the EF-Tu receptor gene *AtEFR* of *Arabidopsis* in tomato, potato, tobacco, wheat, and rice enhanced resistance to different pathogenic bacteria [136,137,138,139,140,141,142]. However, the safety assessment for the efficacy, durability, absence of toxicity, and low environmental impact of these transgenic events are much more difficult than other transgenes. None of them have been authorized for cultivation and market [125].

### 3.2. Resistance to Parasitic Nematodes

Although overexpression of endogenous and exogenous resistance genes, such as the *Mi* from tomato for resistance against *Meloidogyne incognita*, the *pro-1* from sugar beet against *Heterodera schachtii*, the *Gpa-2* from potato against *Globodera allida*, and the *Hero* from tomato against *Globodera rostochiensis*, as well as the genes of cowpea trypsin inhibitor, cystatins, and serine proteases, showed some enhancement to the resistance of the transgenic lines [143]; the most effective strategy of transgenic improvement for resistance to parasitic nematodes is RNAi, which was first discovered in the model non-parasitic nematode *C*. *elegans* and also demonstrated in plant-parasitic nematodes [144,145,146]. Crop plants are transformed by RNAi constructs to express dsRNA molecules with sequences derived from the target genes related to parasitism and the development of nematodes, such as dual oxidase, splicing factor, integrase, cathepsin L cysteine proteinase, proteasome integrity-related protein, signal peptidase, tyrosine phosphatase, neuropeptide, as well as aspartic, serine, cysteine proteases, and effector in *M*. *incognita* [147,148,149,150,151,152,153,154,155,156], *Pratylenchus vulnus* [157], and *Meloidogyne chitwoodi* [158], and protease and cyst formation- and reproduction-associated proteins, as well as three genes associated with reproduction to fitness in *Heterodera glycines* [159,160]. Among them, the effector genes should be a worthwhile group of target genes for in planta RNAi strategy as effectors generally lack high homology with the genes of organisms from other taxa, thereby diminishing the potential for problems related to off-target effects [161].

## 4. Strategy Option of Transgenic Improvement

### 4.1. Three Strategies of Transgenic Manipulation

The heterologous expression of exogenous genes from genetically distant species, overexpression of endogenous genes from the same species or homologous genes from sexually compatible species, and silencing of the expression of suboptimal endogenous, pathogenic, or pest genes by RNAi or CRISPR/Cas are three strategies of transgenic improvement. The first strategy is the transformation by exogenous genes from genetically distant species. The second strategy is the transformation of endogenous genes from the same species or homologous genes from sexually compatible species. The third strategy is silencing the expression of undesirable endogenous or pathogenic and pest genes by RNAi) or CRISPR/Cas technology [162].

### 4.2. Heterologous Expression of Exogenous Genes

The exogenous transgenes were promoted by the constitutive promoters and the possible codon usage bias was usually overcome by codon optimization of the transgene sequences [163,164]. Therefore, their heterologous expression was usually not regulated on the transcriptional level, although the expression levels might be affected by genetic background and growth stage of the recipient cultivars, as well as environmental conditions [77,165,166,167]. The investigations in transgenic insecticidal cotton (r = 0.762, *p* < 0.001) and rice (r = 0.742, *p* < 0.01) show that the accumulation of the Cry protein in leaves is nonlinearly correlated with the heterologous transcription levels of the exogenous *Cry* genes, although varying with growth and development [168,169,170,171]. Therefore, the novel functions conferred by the exogenous transgenes, such as resistance to herbivory insects [74,76,77,78,79,80], usually performed in pathways divergent from the endogenous metabolism of the recipient crops [172,173,174]. This strategy should be hopeful for transgenic improvement for broad-spectrum resistance to pathogenic fungi with the detailed elucidation of the mechanism of the hypersensitive response mediated by the non-host resistant or avirulence genes [175,176,177,178].

### 4.3. Overexpression of Endogenous Genes

Biochemical reactions are reversible and regulated in complex networks [80,179]. The activity of any step enhanced by overexpression of the gene encoding the enzyme may not desirably result in increased flux through the reaction that it catalyzes [77,180]. Moreover, transformation may generate completely new interactions between the transgenes, making them function differently from what is expected. According to the survey carried out by ISAAA (http://www.isaaa.org/gmapprovaldatabase/default.asp, accessed on 22 September 2022), 93 of the 118 singular transgenic events of biotic resistance authorized for cultivation and market were created by the first strategy and transformed by bacterial insect-resistant genes [77,179,180,181], except one exogenous gene from a sexually incompatible plant species was introduced into one event for pyramiding herbicide resistance [84]. The other 25 events were created by the third strategy and transformed by synthetic sequences transcribing antisense or double-stranded RNAs for silencing the expression of housekeeping genes of pathogenic viruses [92,93,94,97,100,101,102,182,183,184,185]. Of course, antibiotic or herbicide-resistant genes from bacteria were also introduced into almost all of these events as selection markers of transformant screening [186]. Although a lot of the literature has documented the improvement of biotic resistance by overexpression of endogenous genes or homologous genes [187,188,189,190,191,192], all of them remain at the testing stage and none of them have been released commercially through safety evaluation. It is questionable whether this strategy will ever be widely used in transgenic improvement for biotic constraints [107].

### 4.4. RNA Interference

RNAi triggered by antisense or double-stranded RNAs described by transformed synthetic DNA sequences is a versatile, effective, safe, and eco-friendly technology for crop protection against viruses and other pathogens as well as herbivory insect and nematodes [71,193,194,195]. Because of the advantages, as it does not produce any functional foreign proteins and targets organisms in a sequence-specific manner, host-delivered RNAi has become a powerful tool of transgenic improvement for biotic constraints [71,193,194,195,196]. Up to now, 25 transgenic events of resistant to pathogenic viruses have been authorized for cultivation and market (Table 2) [93,100,101,102]. In particular, the transgenic papaya cultivars resistant to ringspot virus, that is a devastating disease, have rescued the entire production of papaya [182]. The creation of a topical application of dsRNA using layered double-hydroxide clay nanosheets opens up possibilities to exploit such innovations for specific and combinatorial resistance against herbivory insects and plant parasitic nematodes [197]. However, off-target effects can originate due to sequence similarity between dsRNA and non-target mRNA transcripts resulting in an unintended silence of non-target genes and an unexpected phenotype [198,199,200]. This is an important consideration for biosafety assessment of transgenic events of host-generating RNAi. Therefore, proper designing of the dsRNA sequence to preventing the off-target effects, as well as probable non-target effects, is crucial for the wider employment of RNAi. The advancement of genome sequence data, functional genomics availability, and new bioinformatic tools have become helpful for the precise design of dsRNA sequence [201]. RNAi strategy is also hopeful for transgenic improvement for resistance to pathogenic fungi and bacteria with the detailed elucidation of the crucial factors of pathogenicity [202,203,204].

### 4.5. Gene Edition

After zinc finger nuclease (ZFN) and transcription activator-like effector nuclease (TALEN), CRISPR/Cas technology emerged as a precise, simple, and cost-effective tool of RNA-guided genome editing and is now becoming widely applied to edit the genomes of a diverse range of crop plants [205,206,207,208]. Without incorporating any foreign DNA, it is even proposed as non-transgenic genetic modification fostering public acceptance [209,210]. At the beginning, most studies focused more on the concept proofing of the CRISPR/Cas system [211,212,213,214,215,216]. In recent years, the probable off-target effects caused by the imperfect matches with gRNA and the unpredictable efficiency among different DNA target sites and PAM were effectively reduced with the advances in high attractive sgRNA, high fidelity Cas 9, and transformant screening [206,217]. This system has also been applied to improve crops for biotic resistance, such as overexpressing hypersensitivity-responsive genes of host crops, suppressing effector genes of pathogens and susceptibility genes of host crops, such as virus resistance [112,218,219,220,221,222,223,224,225,226,227,228,229,230,231,232,233]. For example, bacterial blight disease, caused by *Xanthomonas oryzae* pv. *oryzae*, is a major disease that affects rice production. The expression of sucrose-transporter genes SWEET1, SWEET3, and SWEET14 causes disease susceptibility. CRISPR/Cas-editing inhibited the activation of the susceptibility gene (S) of *X*. *oryzae* and prevented its encoding for transcription activator-like effectors (TALEs). The *SWEET* genes could not be activated by the binding of the TALES to their effector binding elements (EBEs) during their promoters and prevented the establishment of host susceptibility [234,235]. In recent years, more and more biotic resistance has been included in the ranks of CRISPR/Cas-mediated improvements (Table 3) [104,208,234,235,236,237,238,239,240,241,242]. However, only a very few events have been in the pipeline of safety assessment up to now [222,224]. Several events have successfully skipped the regulation of government and entered the market because of their safety assurance, and some more events have been in the pipeline of safety assessment [227,228,243]. Recently, CRISPR/Cas13 was characterized as an RNA-guided RNA editing system with high cleavage activities to foreign RNAs [244]. This system can precisely target plant RNA viruses to impart resistance, and even can be multiplexed by designing a streamlined multiple gRNA cassette [105,245]. By this strategy, three transgenic potato lines with broad-spectrum resistance were developed by the introduction of a multiple gRNA cassette of CRSIPR/Cas13 system to target six genes of three potato virus strains [106].

## 5. Conclusions

Transgenic manipulation is a direct and directional approach to improve crops for biotic resistance. The heterologous expression of exogenous genes from genetically distant species, as well as silencing the expression by RNAi and CRISPR/Cas is more effective than overexpression of endogenous genes. Recent advances in RNAi and CRISPR/Cas technologies open up possibilities to improve the resistance of crops to pathogenic bacteria and plant parasitic nematodes, as well as the other biotic constraints, and enable the preclusion of public concerns and engendering of public acceptance.

## Figures and Tables

**Table 1:** Number of transgenic events of insect resistance authorized for cultivation and market.

Crop	Gene Introduced	Doner Species	Target Insect			Released Event
			*Lepidopteran*	*Coleopteran*	*Hemipteran*	
Cotton	*Cry*	*B*. *thuringiensis*	23 (1)		1	25
	*vip3*		1			
	*CpTI*	*V*. *unguiculata*	(1)			
Cowpea	*Cry*	*B*. *thuringiensis*	1			1
Eggplant	*Cry*	*B*. *thuringiensis*	1			1
Maize	*Cry*	*B*. *thuringiensis*	15 (2)	8 (3)		25
	*vip3*	*B*. *thuringiensis*	2 (1)			
	RNAi of *Snf7*	*D*. *virgifera*		(1)		
Potato	*Cry*	*B*. *thuringiensis*		30		30
Rice	*Cry*	*B*. *thuringiensis*	3 (2)			3
Soybean	*Cry*	*B*. *thuringiensis*	4 (2)			4
Sugarcane	*Cry*	*B*. *thuringiensis*	3			3
Tomato	*Cry*	*B*. *thuringiensis*	1			1
Total			54	38	1	93

Note: The brackets indicate the numbers of the genes introduced for pyramiding.

**Table 2:** Number of RNAi transgenic events of viral resistance authorized for cultivation and market.

Crop	Gene Introduced	Target Virus	Released Event
Bean	Replicase gene *bgmv_ac1*	Bean golden mosaic virus	1
Papaya	Coat protein gene *prsv_cp*	Papaya ringspot virus	3
	Replicase gene *prsv_rep*		1
Plum	Coat protein gene *ppv_cp*	Plum pox virus	1
Potato	Coat protein gene *pvy_cp*	Potato virus Y	8
	Replicase gene *plrv_orf1*	Potato leaf roll virus	7
	Helicase gene *plrv_orf2*		(7)
Squash	Coat protein gene *zymv_cp*	Zucchini yellow mosaic virus	2
	Coat protein gene *cmv_cp*	Cucumber mosaic virus	[1]
	Coat protein gene *wmv_cp*	Watermelon mosaic virus 2	[2]
Sweet pepper	Coat protein gene *cmv_cp*	Cucumber mosaic virus	1
Tomato	Coat protein gene *cmv_cp*	Cucumber mosaic virus	1
Total			25

Note: The parentheses and square brackets indicate the numbers of the genes introduced for resistance pyramiding or broadening, respectively.

**Table 3:** CRISPR-Cas edited improvement of crops for disease resistance.

Crop	Gene Modified	Resistance Improved	Reference
Citrus	*CsLOB1*	Citrus canker	[236]
Cassava	*nCBP-1, nCBP-2*	Cassava brown streak disease	[237]
Cucumber	*eIF4E*	Cucumber vein yellowing virus	[238]
Grape	*VvWRKY52*	*Botrytis cinerea*	[239]
Rice	*Bsr-k1*	Broad spectrum resistance	[234]
	*OsSWEET11, 13, 14*	Bacterial blight	[235]
Tomato	*Pmr4*	Powdery mildew	[240]
	*Jaz2*	Bacterial speck disease	[241]
Wheat	*TaMlo-A1, -B1, -D1*	Powdery mildew	[220]
	*TaEdr1* (three homologs)		[242]

## References

[1] Oerke E.C. (2006). Crop losses to pests. J. Agric. Sci..

[2] Gimenez E., Salinas M., Manzano-Agugliaro F. (2018). Worldwide research on plant defense against biotic stresses as improvement for sustainable agriculture. Sustainability.

[3] McKenna T.P., Koziol L., Bever J.D., Crews T.E., Sikes B.A. (2020). Abiotic and biotic context dependency of perennial crop yield. PLoS ONE.

[4] Nazarov P.A., Baleev D.N., Ivanova M.I., Sokolova L.M., Karakozova M.V. (2020). Infectious plant diseases: Etiology, current status, problems and prospects in plant protection. Acta Nat..

[5] Schumann G.L., D’Arcy C.J. (2006). Essential Plant Pathology.

[6] Falk B.W., Nouri S. (2020). Special issue: Plant virus pathogenesis and disease control. Viruses.

[7] Douglas A.E. (2018). Strategies for enhanced crop resistance to insect pests. Annu. Rev. Plant Biol..

[8] Jones J.T., Haegeman A., Danchin E.G.J., Gaur H.S., Helder J., Jones M.G.K., Kikuchi T., Manzanilla-López R., Palomares-Rius J.E., Wesemael W.M. (2013). Top 10 plant-parasitic nematodes in molecular plant pathology. Mol. Plant Pathol..

[9] Elling A.A. (2013). Major emerging problems with minor *Meloidogyne* species. Phytopathology.

[10] Ali M.A., Azeem F., Abbas A., Joyia F.A., Li H., Dababat A.A. (2017). Transgenic strategies for enhancement of nematode resistance in plants. Front. Plant Sci..

[11] Jones D.G., Dangl L. (2006). The plant immune system. Nature.

[12] Schreiber K., Desveaux D. (2008). Message in a bottle: Chemical biology of induced disease resistance in plants. Plant Pathol. J..

[13] Spoel S.H., Dong X. (2012). How do plants achieve immunity? Defence without specialized immune cells. Nat. Rev. Immunol..

[14] Monaghan J., Zipfel C. (2012). Plant pattern recognition receptor complexes at the plasma membrane. Curr. Opin. Plant Biol..

[15] Muthamilarasan M., Prasad M. (2013). Plant innate immunity: An updated insight into defense mechanism. J. Biosci..

[16] Savary S., Willocquet L., Pethybridge S.J., Esker P., McRoberts N., Nelson A. (2019). The global burden of pathogens and pests on major food crops. Nat. Ecol. Evol..

[17] Savary S., Willocquet L. (2020). Modeling the impact of crop diseases on global food security. Ann. Rev. Phytopathol..

[18] Hampf A.C., Nendel C., Strey S., Strey R. (2021). Biotic yield losses in the Southern Amazon, Brazil: Making use of smartphone–assisted plant disease diagnosis data. Front. Plant Sci..

[19] Fontana D.C., de Paula S., Torres A.G., de Souza V.H.M., Pascholati S.F., Schmidt D., Dourado Neto D. (2021). Endophytic fungi: Biological control and induced resistance to phytopathogens and abiotic stresses. Pathogens.

[20] Kim C., Cho W., Kim H. (2000). Yield loss of spring Chinese cabbage as affected by infection time of clubroot disease in fields. Plant Dis. Res..

[21] Shukla A.K. (2005). Estimation of yield losses to Indian mustard (*Brassica juncea*) due to Sclerotinia stem rot. J. Phytol. Res..

[22] Poveda J., Francisco M., Cartea M.E., Velasco P. (2020). Development of transgenic *Brassica* crops against biotic stresses caused by pathogens and arthropod pests. Plants.

[23] Barrett C.B. (2021). Overcoming global food security challenges through science and solidarity. Am. J. Agric. Econ..

[24] Roth M.G., Webster R.W., Mueller D.S., Chilvers M.I., Faske T.R., Mathew F.M., Bradley C.A., Damicone J.P., Kabbage M., Smith D.L. (2020). Integrated management of important soybean pathogens of the United States in changing climate. J. Integr. Pest Manag..

[25] Yin K., Qiu J.L. (2019). Genome editing for plant disease resistance: Applications and perspectives. Philos. Trans. R. Soc. Lond. B Biol. Sci..

[26] Tester M., Langridge P. (2010). Breeding technologies to increase crop production in a changing world. Science.

[27] Nelson R., Wiesner-Hanks T., Wisser R., Balint-Kurti P. (2018). Navigating complexity to breed disease-resistant crops. Nat. Rev. Genet..

[28] Sharma A., Abrahamian P., Carvalho R., Choudhary M., Paret M.L., Vallad G.E., Jones J.B. (2022). Future of bacterial disease management in crop production. Annu. Rev. Phytopathol..

[29] Li C. (2020). Breeding crops by design for future agriculture. J. Zhejiang Univ. Sci. B.

[30] Roelfs A.P. (1988). Genetic control of phenotypes in wheat stem rust. Ann. Rev. Phytopathol..

[31] Elshafei A.A., Motawei M.I., Esmail R.M., Al-Doss A.A., Hussien A.M., Ibrahim E.I., Amer. M.A. (2021). Molecular breeding for rust resistance in wheat genotypes. Mol. Biol. Rep..

[32] Denes T.E., Molnar I., Rakosy-Tican E. (2015). New insights into the interaction between cultivated potato and *Phytophthora infestans*. Stud. Univ. Babes-Bolyai Biol..

[33] Joosten M.H.A.J., Cozijnsen T.J., de Wit P.J.G.M. (1994). Host resistance to fungal tomato pathogen lost by a single base pair change in an avirulence gene. Nature.

[34] Gassmann W., Dahlbeck D., Chesnokova O., Minsavage G.V., Jones J.B., Staskawicz B.J. (2000). Molecular evolution of virulence in natural field strains of *Xanthomonas campestris* pv. vesicatoria. J. Bacteriol..

[35] Stukenbrock E.H., McDonald B.A. (2009). Population genetics of fungal and oomycete effectors involved in gene-for-gene interactions. Mol. Plant Microbe Interact..

[36] Dodds P., Thrall P. (2009). Recognition events and host-pathogen co-evolution in gene-for-gene resistance to flax rust. Funct. Plant Biol..

[37] Solomon-Blackburn R.M. (1998). Progress in breeding potatoes for resistance to virus diseases. Asp. Appl. Biol..

[38] Liu J., Liu D., Tao W., Li W., Wang S., Chen P., Cheng S., Gao D. (2000). Molecular marker-facilitated pyramiding of different genes for powdery mildew resistance in wheat. Plant Breed..

[39] Richardson K.L., Vales M.I., Kling J.G., Mundt C.C., Hayes P.M. (2006). Pyramiding and dissecting disease resistance QTL to barley stripe rust. Theor. Appl. Genet..

[40] Saghai Maroof M.A., Jeong S.C., Gunduz I., Tucker D.M., Buss G.R., Tolin S.A. (2008). Pyramiding of soybean mosaic virus resistance genes by marker-assisted selection. Crop Sci..

[41] Keane P.J., Cumagun C.J. (2012). Horizontal or Generalized Resistance to Pathogens in Plants. Plant Pathology.

[42] Galvez L.C., Banerjee J., Pinar H., Mitra A. (2014). Engineered plant virus resistance. Plant Sci..

[43] Mendes B.J., Filho F.M., Farias P., Benedito V. (2001). Citrus somatic hybridization with potential for improved blight and CTV resistance. In Vitro Cell. Dev. Biol. Plant.

[44] Collard B.C., Mackill D.J. (2008). Marker-assisted selection: An approach for precision plant breeding in the twenty-first century. Philos. Trans. R. Soc. Lond. Ser. B Biol. Sci..

[45] Asea G., Vivek B.S., Bigirwa G., Lipps P.E., Pratt R.C. (2009). Validation of consensus quantitative trait loci associated with resistance to multiple foliar pathogens of maize. Phytopathology.

[46] Bohar R., Chitkineni A., Varshney R.K. (2020). Genetic molecular markers to accelerate genetic gains in crops. Biotechniques.

[47] Webster R.W., Roth M.G., Reed H., Mueller B., Groves C.L., McCaghey M., Chilvers M.I., Mueller D.S., Kabbage M., Smith D.L. (2021). Identification of soybean (*Glycine max*) check lines for evaluating genetic resistance to sclerotinia stem rot. Plant Dis..

[48] Foria S., Magris G., Jurman I., Schwope R., de Candido M., de Luca E., Ivanisevic D., Morgante M., Di Gaspero G. (2022). Extent of wild-to-crop interspecific introgression in grapevine (*Vitis inifera*) as a consequence of resistance breeding and implications for the crop species definition. Hortic. Res..

[49] Purankar M.V., Nikam A.A., Devarumath R.M., Suprasanna P. (2022). Radiation induced mutagenesis, physio-biochemical profiling and field evaluation of mutants in sugarcane Cv. CoM 0265. Int. J. Radiat. Biol..

[50] Batsa B.K., Sharma R.C., Rai S.N. (2005). Identifying resistance to banded leaf and sheath blight of maize. Indian Phytopathol..

[51] Kang B.C., Yeam I., Jahn M.M. (2005). Genetics of plant virus resistance. Annu. Rev. Phytopathol..

[52] Holbein J., Grundler F.M., Siddique S. (2016). Plant basal resistance to nematodes: An update. J. Exp. Bot..

[53] Preston G.M. (2017). Profiling the extended phenotype of plant pathogens: Challenges in bacterial molecular plant pathology. Mol. Plant Pathol..

[54] Aktar W., Sengupta D., Chowdhur Y.A. (2009). Impact of pesticides use in agriculture: Their benefits and hazards. Interdiscip. Toxicol..

[55] Damalas C.A., Eleftherohorinos I.G. (2011). Pesticide exposure, safety issues, and risk assessment indicators. Int. J. Environ. Res. Public Health.

[56] Birkett M.A., Pickett J.A. (2014). Prospects of genetic engineering for robust insect resistance. Curr. Opin. Plant Biol..

[57] Sharma A., Kumar V., Shahzad B., Tanveer M., Sidhu G.P.S., Handa N., Kohli S.K., Yadav P., Bali A.S., Parihar R.D. (2019). Worldwide pesticide usage and its impacts on ecosystem. SN Appl. Sci..

[58] Castle S., Palumbo J., Prabhaker N. (2009). Newer insecticides for plant virus disease management. Virus Res..

[59] Dababat A., Imren M., Erginbas-Orakci G., Ashrafi S., Yavuzaslanoglu E., Toktay H., Pariyar S.R., Elekcioglu H.I., Morgounov A., Mekete T. (2015). The importance and management strategies of cereal cyst nematodes, *Heterodera* spp., in Turkey. Euphytica.

[60] Bardin M., Ajouzm S., Comby M., Lopez-Ferber M., Graillot B., Siegwart M., Nicot P.C. (2015). Is the efficacy of biological control against plant diseases likely to be more durable than that of chemical pesticides?. Front. Plant Sci..

[61] He D.C., He M.H., Amalin. D.M., Liu W., Alvindia D.G., Zhan J. (2021). Biological control of plant diseases: An evolutionary and eco-economic consideration. Pathogens.

[62] Raymond Park J., Mcfarlane I., Hartley Phipps R., Ceddia G. (2011). The role of transgenic crops in sustainable development. Plant Biotechnol. J..

[63] Kamthan A., Chaudhuri A., Kamthan M., Datta A. (2016). Genetically modified (GM) crops: Milestones and new advances in crop improvement. Theor. App. Genet..

[64] Ahmar S., Gill R.A., Jung K.H., Faheem A., Qasim M.U., Mubeen M., Zhou W. (2020). Conventional and molecular techniques from simple breeding to speed breeding in crop plants: Recent advances and future outlook. Int. J. Mol. Sci..

[65] Eckardt N.A., Cominelli E., Galbiati M., Tonelli C. (2009). The future of science: Food and water for life. Plant Cell.

[66] Farre G., Ramessar K., Twyman R.M., Capell T., Christou P. (2010). The humanitarian impact of plant biotechnology: Recent breakthroughs vs bottlenecks for adoption. Curr. Opin. Plant Biol..

[67] Anjanappa R.B., Gruissem W. (2021). Current progress and challenges in crop genetic transformation. J. Plant Physiol..

[68] Georges F., Ray H. (2017). Genome editing of crops: A renewed opportunity for food security. GM Crops Food.

[69] Zhang J., Khan S.A., Heckel D.G., Bock R. (2017). Next-generation insect-resistant plants: RNAi-mediated crop protection. Trends Biotechnol..

[70] Mamta B., Rajam M.V. (2017). RNAi technology: A new platform for crop pest control. Physiol. Mol. Biol. Plants.

[71] Hernández-Soto A., Chacón-Cerdas R. (2021). RNAi crop protection advances. Int. J. Mol. Sci..

[72] Xu R., Zheng Z., Jiao G. (2014). Safety assessment and detection methods of genetically modified organisms. Recent Pat. Food Nutr. Agric..

[73] Tilgam J., Kumar K., Jayaswal D., Choudhury S., Kumar A., Jayaswall K., Saxena A.K. (2021). Success of microbial genes based transgenic crops: Bt and beyond Bt. Mol. Biol. Rep..

[74] Smyth S., Phillips P.W.B., Castle D. (2014). Benefits of genetically modified herbicide tolerant canola in Western Canada. Int. J. Biotechnol..

[75] Klumper W., Qaim M. (2014). A meta-analysis of the impacts of genetically modified crops. PLoS ONE.

[76] Pray C., Ma D., Huang J., Qiao F. (2001). Impact of Bt cotton in China. World Dev..

[77] Showalter A.M., Heuberger S., Tabashnik B.E., Carriere Y., Coates B. (2009). A primer for using transgenic insecticidal cotton in developing countries. J. Insect Sci..

[78] Blanco C.A. (2012). *Heliothis virescens* and Bt cotton in the United States. GM Crops Food.

[79] Rocha-Munive M.G., Soberon M., Castaneda S., Niaves E., Scheinvar E., Eguiarte L.E., Mota-Sánchez D., Rosales-Robles E., Nava-Camberos U., Martinez-Carrillo J.L. (2018). Evaluation of the impact of genetically modified cotton after 20 years of cultivation in Mexico. Front. Bioeng. Biotechnol..

[80] Ghareyazie B., Alinia F., Menguito C.A., Rubia L.G., de Palma J.M., Liwanag E.A., Cohen M.B., Khush G.S., Bennett J. (1997). Enhanced resistance to two stem borers in an aromatic rice containing a synthetic *cryIA*(b) gene. Mol. Breed..

[81] Xie Z.W., Luo M.J., Xu W.F., Chi C.W. (1997). Two reactive site locations and structure-function study of the arrowhead proteinase inhibitors, A and B, using mutagenesis. Biochemistry.

[82] Hu J.J., Tian Y.C., Han Y.F., Li Y., Zhang B.E. (2001). Field evaluation of insect-resistant transgenic *Populus nigra* trees. Euphytica.

[83] Cui J., Luo J., van der Werf W., Ma Y., Xia J. (2011). Effect of pyramiding *Bt* and *CpTI* genes on resistance of cotton to *Helicoverpa armigera* (*Lepidoptera*: *Noctuidae*) under laboratory and field conditions. J. Econ. Entomol..

[84] Ramaseshadri P., Segers G., Flannagan R., Wiggins E., Clinton W., Ilagan O., McNulty B., Clark T., Bolognesi R. (2013). Physiological and cellular responses caused by RNAi-mediated suppression of *Snf7* orthologue in western corn rootworm (*Diabrotica virgifera virgifera*) larvae. PLoS ONE.

[85] Sanford J.C., Johnston S.A. (1985). The concept of parasite-derived resistance- deriving resistance genes from the parasite’s own genome. J. Theor. Biol..

[86] Abel P.P., Nelson R.S., De B., Hoffmann N., Rogers S.G., Fraley R.T., Beachy R.N. (1986). Delay of disease development in transgenic plants that express the tobacco mosaic virus coat protein gene. Science.

[87] Tennant P.F., Gonsalves C., Ling K.S., Fitch M., Manshardt R., Slightom L.J., Gonsalves D. (1994). Differential protection against papaya ringspot virus isolates in coat protein gene transgenic papaya and classically cross-protected papaya. Phytopathology.

[88] Mundembe R., Matibiri A., Sithole-Niang I. (2009). Transgenic plants expressing the coat protein gene of cowpea aphid-borne mosaic potyvirus predominantly convey the delayed symptom development phenotype. Afr. J. Biotechnol..

[89] Golemboski D.B., Lomonossoff G.P., Zaitlin M. (1990). Plants transformed with a tobacco mosaic virus nonstructural gene sequence are resistant to the virus. Proc. Natl. Acad. Sci. USA..

[90] Fire A., Xu S., Montgomery M.K., Kostas S.A., Driver S.E., Mello C.C. (1998). Potent and specific genetic interference by double stranded RNA in *Caenorhabditis elegans*. Nature.

[91] Prins M., Laimer M., Noris E., Schubert J., Wassenegger M., Tepfer M. (2008). Strategies for antiviral resistance in transgenic plants. Mol. Plant Pathol..

[92] Chen Z., Gu H., Li Y., Su Y., Wu P., Jiang Z., Ming X., Tian J., Pan N., Qu L.J. (2003). Safety assessment for genetically modified sweet pepper and tomato. Toxicology.

[93] Tennant P., Souza M.T., Fitch M.M., Manshardt R.M., Slightom J.L. (2005). Line 63-1: A new virus-resistant transgenic papaya. Hortscience.

[94] Wu Z., Mo C., Zhang S., Li H. (2018). Characterization of papaya ringspot virus isolates infecting transgenic papaya ‘Huanong No.1’ in South China. Sci. Rep..

[95] Brunetti A., Tavazza M., Noris E., Tavazza R., Caciagli P., Ancora G., Crespi S., Accotto G.P. (1997). High expression of truncated viral rep protein confers resistance to tomato yellow leaf curl virus in transgenic tomato plants. Mol. Plant Microbe Interact..

[96] Faria J.C., Albino M.M.C., Dias B.B.A., Cançado L.J., Cunha N.B.D., Silva L.D.M., Vianna G.R., Aragão F.J.L. (2006). Partial resistance to bean golden mosaic virus in a transgenic common bean (*Phaseolus vulgaris* L.) line expressing a mutated *rep* gene. Plant Sci..

[97] Aragao F.J.L., Nogueira E.O.P.L., Tinoco M.L.P., Faria J.C. (2013). Molecular characterization of the first commercial transgenic common bean immune to the bean golden mosaic virus. J. Biotechnol..

[98] Lapidot M., Gafny R., Ding B., Wolf S., Lucas W.J., Beachy R.N. (1993). A dysfunctional movement protein of tobacco mosaic virus that partially modifies the plasmodesmata and limits virus spread in transgenic plants. Plant J..

[99] Cooper B., Lapidot M., Heick J.A., Dodds J.A., Beachy R.N. (1995). A defective movement protein of TMV in transgenic plants confers resistance to multiple viruses whereas the functional analog increases susceptibility. Virology.

[100] Carvalho J.L., De Oliveira Santos J., Conte C., Pacheco S., Nogueira E.O., Souza T.L., Faria J.C., Aragão F.J. (2015). Comparative analysis of nutritional compositions of transgenic RNAi-mediated virus-resistant bean (event EMB-PV051-1) with its non-transgenic counterpart. Transgenic Res..

[101] Borah M., Berbati M., Reppa C., Holeva M., Nath P.D., Voloudakis A. (2018). RNA-based vaccination of Bhut Jolokia pepper (*Capsicum chinense* Jacq.) against cucumber mosaic virus. Virusdisease.

[102] Callahan A.M., Dardick C.D., Scorza R. (2019). Multilocation comparison of fruit composition for ‘HoneySweet’, an RNAi based plum pox virus resistant plum. PLoS ONE.

[103] Halterman D.A., Kramer L.C., Wielgus S., Jiang J. (2008). Performance of transgenic potato containing the late blight resistance gene RB. Plant Dis..

[104] Zaidi S.S., Tashkandi M., Mansoor S., Mahfouz M.M. (2016). Engineering plant immunity: Using CRISPR/Cas9 to generate virus resistance. Front. Plant Sci..

[105] Khan M.Z., Amin I., Hameed A., Mansoor S. (2018). CRISPR-Cas13a: Prospects for plant virus resistance. Trends Biotechnol..

[106] Noureen A., Khan M.Z., Amin I., Zainab T., Ahmad N., Haider S., Mansoor S. (2022). Broad-spectrum resistance against multiple PVY strains by CRSIPR/Cas13 system in *Solanum tuberosum* crop. GM Crops Food.

[107] Stuiver M.H., Custers J.H. (2001). Engineering disease resistance in plants. Nature.

[108] Wally O., Punja Z.K. (2010). Genetic engineering for increasing fungal and bacterial disease resistance in crop plants. GM Crops.

[109] Hwang B.H., Bae H., Lim H.S., Kim K.B., Kim S.J., Im M.H., Park B.S., Kim J. (2010). Overexpression of polygalacturonase-inhibiting protein 2 (PGIP2) of Chinese cabbage (*Brassica rapa* ssp. *pekinensis*) increased resistance to the bacterial pathogen *Pectobacterium carotovorum* ssp. *carotovorum*. Plant Cell Tissue Organ Cult..

[110] Wang Z., Fang H., Chen Y., Chen K., Li G., Gu S., Tan X. (2014). Overexpression of *BnWRKY33* in oilseed rape enhances resistance to *Sclerotinia sclerotiorum*. Mol. Plant Pathol..

[111] Zhang Y., Huai D., Yang Q., Cheng Y., Ma M., Kliebenstein D.J., Zhou Y. (2015). Overexpression of three glucosinolate iosynthesis genes in *Brassica napus* identifies enhanced resistance to *Sclerotinia sclerotiorum* and *Botrytis cinerea*. PLoS ONE.

[112] Fang Y., Tyler B.M. (2016). Efficient disruption and replacement of an effector gene in the oomycete *Phytophthora sojae* using CRISPR/Cas9. Mol. Plant Pathol..

[113] Ali S., Mir Z.A., Tyagi A., Mehari H., Meena R.P., Bhat A., Yadav P., Papalou P., Rawat S., Grover A. (2017). Overexpression of NPR1 in *Brassica juncea* confers road spectrum resistance to fungal pathogens. Front. Plant Sci..

[114] Cui J., Jiang N., Zhou X., Hou X., Yang G., Meng J., Luan Y. (2018). Tomato MYB49 enhances resistance to *Phytophthora infestans* and tolerance to water deficit and salt stress. Planta.

[115] Liu T., Chen T., Kan J., Yao Y., Guo D., Yang Y., Ling X., Wang J., Zhang B. (2022). The GhMYB36 transcription factor confers resistance to biotic and abiotic stress by enhancing *PR1* gene expression in plants. Plant Biotechnol. J..

[116] Anand A., Zhou T., Trick H.N., Gill B.S., Bockus W.W., Muthukrishnan S. (2003). Greenhouse and field testing of transgenic wheat plants stably expressing genes for thaumatin-like protein, chitinase and glucanase against *Fusarium graminearum*. J. Exp. Bot..

[117] Yang S., Gao M., Xu C., Gao J., Deshpande S., Lin S., Roe B.A., Zhu H. (2008). Alfalfa benefits from *Medicago truncatula*: The *RCT1* gene from *M. truncatula* confers broad spectrum resistance to anthracnose in alfalfa. Proc. Natl. Acad. Sci. USA.

[118] Alexander D., Goodman R.M., Gut-Rella M., Glascock C., Weymann K., Friedrich L., Maddox D., Ahl-Goy P., Luntz T., Ward E. (1993). Increased tolerance to two oomycete pathogens in transgenic tobacco expressing pathogenesis-related protein 1a. Proc. Natl. Acad. Sci. USA.

[119] Doares S.H., Narvaez-Vazquez J., Conconi A., Ryan C.A. (1995). Salicylic acid inhibits synthesis of proteinase inhibitors in tomato leaves induced by systemin and jasmonic acid. Plant Physiol..

[120] Albert I., Böhm H., Albert M., Feiler C.E., Imkampe J., Wallmeroth N., Brancato C., Raaymakers T.M., Oome S., Zhang H. (2015). An RLP23-SOBIR1-BAK1 complex mediates NLP-triggered immunity. Nat. Plants.

[121] Song Y., Liu L., Wang Y., Valkenburg D.J., Zhang X., Zhu L., Thomma B.P.H.J. (2018). Transfer of tomato immune receptor *Ve1* confers Ave1-dependent *Verticillium* resistance in tobacco and cotton. Plant Biotechnol. J..

[122] Du J., Verzaux E., Chaparro-Garcia A., Bijsterbosch G., Keizer L.C.P., Zhou J., Liebrand T.W.H., Xie C., Govers F., Robatzek S. (2015). Elicitin recognition confers enhanced resistance to *Phytophthora infestans* in potato. Nat. Plants.

[123] Foster S.J., Park T.H., Pel M., Brigneti G., Sliwka J., Jagger L., van der Vossen E., Jones J.D. (2009). Rpi-vnt1.1, a Tm-2(2) homolog from *Solanum venturii*, confers resistance to potato late blight. Mol. Plant Microbe Interact..

[124] Luo M.L., Xie S., Chakraborty A., Wang O., Matny M., Jugovich J.A., Kolmer T., Richardson D., Bhatt M., Hoque M. (2021). A five-transgene cassette confers broad-spectrum resistance to a fungal rust pathogen in wheat. Nat. Biotechnol..

[125] Mourgues F., Brisset M.N., Chevreau E. (1998). Strategies to improve plant resistance to bacterial diseases through genetic engineering. Trends Biotechnol..

[126] Zhou Y.L., Xu J.L., Zhou S.C., Yu J., Xie X.W., Xu M.R., Sun Y., Zhu L.H., Fu B.Y., Gao Y.M. (2009). Pyramiding *Xa23* and *Rxo1* for resistance to two bacterial diseases into an elite indica rice variety using molecular approaches. Mol. Breed..

[127] Jaynes J.M., Nagpala P., Destéfano-Beltrán L., Huang J.H., Kim J.H., Denny T., Cetiner S. (1993). Expression of a cecropin B lytic peptide analog in transgenic tobacco confers enhanced resistance to bacterial wilt caused by *Pseudomonas solanacearum*. Plant Sci..

[128] Huang Y., Nordeen R.O.M.D., Di M., Owens L.D., Mcbeath J.H. (1997). Expression of an engineered cecropin gene cassette in transgenic tobacco plants confers disease resistance to *Pseudomonas syringae* pv. tabaci. Phytopathology.

[129] Mitra A., Zhang Z. (1994). Expression of a human lactoferrin cDNA in tobacco cells produces antibacterial protein(s). Plant Physiol..

[130] Trudel J., Potvin C., Asselin A. (1995). Secreted hen lysozyme in transgenic tobacco: Recovery of bound enzyme and in vitro growth inhibition of plant pathogens. Plant Sci..

[131] Nakajima H., Muranaka T., Ishige F., Akutsu K., Oeda K. (1997). Fungal and bacterial disease resistance in transgenic plants expressing human lysozyme. Plant Cell Rep..

[132] Zhang Z., Coyne D.P., Vidaver A.K., Mitra A. (1998). Expression of human lactoferrin cDNA confers resistance to *Ralstonia solanacearum* in transgenic tobacco plants. Phytopathology.

[133] Rivero M., Furman N., Mencacci N., Picca P., Toum L., Lentz E., Bravo-Almonacid F., Mentaberry A. (2012). Stacking of antimicrobial genes in potato transgenic plants confers increased resistance to bacterial and fungal pathogens. J. Biotechnol..

[134] Lakshman D.K., Natarajan S., Mandal S., Mitra A. (2013). Lactoferrin-derived resistance against plant pathogens in transgenic plants. J. Agric. Food Chem..

[135] Maleck K., Levine A., Eulgem T., Morgan A., Schmid J., Lawton K.A., Dangl J.L., Dietrich R.A. (2000). The transcriptome of *Arabidopsis thaliana* during systemic acquired resistance. Nat. Genet..

[136] Zipfel C., Kunze G., Chinchilla D., Caniard A., Jones J.D.G., Boller T., Felix G. (2006). Perception of the bacterial PAMP EF-Tu by the receptor EFR restricts agrobacterium-mediated transformation. Cell.

[137] Lacombe S., Rougon-Cardoso A., Sherwood E., Peeters N., Dahlbeck D., van Esse H.P., Smoker M., Rallapalli G., Thomma B.P.H.J., Staskawicz B. (2010). Interfamily transfer of a plant pattern-recognition receptor confers broad-spectrum bacterial resistance. Nat. Biotechnol..

[138] Schwessinger B., Bahar O., Thomas N., Holton N., Nekrasov V., Ruan D., Canlas P.E., Daudi A., Petzold C.J., Singan V.R. (2015). Transgenic expression of the dicotyledonous pattern recognition receptor EFR in rice leads to ligand-dependent activation of defense responses. PLoS Pathog..

[139] Schoonbeek H., Wang H.H., Stefanato F.L., Craze M., Bowden S., Wallington E., Zipfel C., Ridout C.J. (2015). *Arabidopsis* EF-Tu receptor enhances bacterial disease resistance in transgenic wheat. New Phytol..

[140] Lu F., Wang H., Wang S., Jiang W., Shan C., Li B., Yang J., Zhang S., Sun W. (2015). Enhancement of innate immune system in monocot rice by transferring the dicotyledonous elongation factor Tu receptor EFR: EFR confers bacterial disease resistance in monocot rice. J. Integr. Plant. Biol..

[141] Boschi F., Schvartzman C., Murchio S., Ferreira V., Siri M.I., Galván G.A., Smoker M., Stransfeld L., Zipfel C., Vilaró F.L. (2017). Enhanced bacterial wilt resistance in potato through expression of *Arabidopsis* EFR and introgression of quantitative resistance from *Solanum commersonii*. Front. Plant Sci..

[142] Pfeilmeier S., George J., Morel A., Roy S., Smoker M., Stransfeld L., Downie J.A., Peeters N., Malone J.G., Zipfel C. (2019). Expression of the *Arabidopsis thaliana* immune receptor EFR in *Medicago truncatula* reduces infection by a root pathogenic bacterium, but not nitrogen-fixing rhizobial symbiosis. Plant Biotechnol. J..

[143] Lilley C.J., Devlin F., Urwin P.E., Atkinson H.J. (1999). Parasitic nematodes, proteinases and transgenic plants. Parasitol. Today.

[144] Bakhetia M., Charlton W.L., Urwin P.E., McPherson M.J., Atkinson H.J. (2005). RNA interference and plant parasitic nematodes. Trends Plant Sci..

[145] Rosso M.N., Dubrana M.P., Cimbolini N., Jaubert S., Abad P. (2005). Application of RNA interference to root-knot nematode genes encoding esophageal gland proteins. Mol. Plant Microbe Interact..

[146] Gheysen G., Kyndt T., Haegeman A., Joseph S., Remy S., Swennen R. (2010). RNAi for research and applications in plant-nematode interactions. In Vitro Cell. Dev. Biol. Anim..

[147] Bakhetia M., Charlton W., Atkinson H.J., McPherson M.J. (2005). RNA interference of dual oxidase in the plant nematode *Meloidogyne incognita*. Mol. Plant Microb. Interact..

[148] Yadav B.C., Veluthambi K., Subramaniam K. (2006). Host generated double stranded RNA induces RNAi in plant parasitic nematodes and protects the host from infection. Mol. Biochem. Parasitol..

[149] Shingles J., Lilley C.J., Atkinson H.J., Urwin P.E. (2007). *Meloidogyne incognita*: Molecular and biochemical characterisation of a cathepsin L cysteine proteinase and the effect on parasitism following RNAi. Exp. Parasitol..

[150] Charlton W.L., Harel H.Y.M., Bakhetia M., Hibbard J.K., Atkinson H.J., Mcpherson M.J. (2010). Additive effects of plant expressed double-stranded RNAs on root-knot nematode development. Int. J. Parasitol..

[151] Ibrahim H.M., Alkharouf N.W., Meyer S.L., Aly M.A., Gamal E.A.K. (2011). Post-transcriptional gene silencing of root-knot nematode in transformed soybean roots. Exp. Parasitol..

[152] Niu J.H., Jian H., Xu J., Chen C., Guo Q. (2012). RNAi silencing of the *Meloidogyne incognita Rpn7* gene reduces nematode parasitic success. Euro. J. Plant Pathol..

[153] Papolu P.K., Gantasala N.P., Kamaraju D., Banakar P., Sreevathsa R., Rao U. (2013). Utility of host delivered RNAi of two FMRF amide like peptides, flp-14 and flp-18, for the management of root-knot nematode, *Meloidogyne incognita*. PLoS ONE.

[154] Antonino de Souza J.D., Ramos Coelho R., Tristan Lourenço I., da Rocha Fragoso R., Barbosa Viana A.A., Lima Pepino de Macedo L., Mattar da Silva M.C., Gomes Carneiro R.M., Engler G., de Almeida-Engler J. (2013). Knocking- down *Meloidogyne incognita* proteases by plant-delivered dsRNA has negative pleiotropic effect on nematode vigor. PLoS ONE.

[155] Dutta T.K., Papolu P.K., Banakar P., Choudhary D., Sirohi A., Rao U. (2015). Tomato transgenic plants expressing hairpin construct of a nematode protease gene conferred enhanced resistance to root-knot nematodes. Front. Microbiol..

[156] Dutta T.K., Banakar P., Rao U. (2015). The status of RNAi-based transgenic research in plant nematology. Front. Microbiol..

[157] Walawage S.L., Britton M.T., Leslie C.A., Uratsu S.L., Li Y., Dandekar M. (2013). Stacking resistance to crown gall and nematodes in walnut rootstocks. BMC Genomics.

[158] Dinh P.T.Y., Zhang L., Brown C.R., Elling A.A. (2014). Plant-mediated RNA interference of effector gene *Mc16D10L* confers resistance against *Meloidogyne chitwoodi* in diverse genetic backgrounds of potato and reduces pathogenicity of nematode offspring. Nematology.

[159] Klink V.P., Kim K.H., Martins V., Macdonald M.H., Beard H.S., Alkharouf N.W., Lee S.K., Park S.C., Matthews B.F. (2009). A correlation between host-mediated expression of parasite genes as tandem inverted repeats and abrogation of development of female *Heterodera glycines* cyst formation during infection of *Glycine max*. Planta.

[160] Li J., Todd T.C., Oakley T.R., Lee J.L., Trick H.N. (2010). Host-derived suppression of nematode reproductive and fitness genes decreases fecundity of *Heterodera glycines Ichinohe*. Planta.

[161] Danchin E.G.J., Arguel M.J., Campan-Fournier A., Perfus-Barbeoch L., Magliano M., Rosso M.N., Da Rocha M., Da Silva C., Nottet N., Labadie K. (2013). Identification of novel target genes for safer and more specific control of root-knot nematodes from a pan-genome mining. PLoS Pathogen..

[162] Yu H., Yang Q., Fu F., Li W. (2022). Three strategies of transgenic manipulation for crop improvement. Front. Plant Sci..

[163] Liu D. (2009). Design of gene constructs for transgenic maize. Meth. Mol. Biol..

[164] Parvathy S.T., Udayasuriyan V., Bhadana V. (2022). Codon usage bias. Mol. Biol. Rep..

[165] Adamczyk J.J., Meredith W.R. (2004). Genetic basis for variability of *Cry1Ac* expression among commercial transgenic *Bacillus thuringiensis* (*Bt*) cotton cultivars in the United States. J. Cotton Sci..

[166] Poongothai S., Iiavarasan R., Karrunakaran C.M. (2010). *Cry1Ac* levels and biochemical variations in *Bt* cotton as influenced by tissue maturity and senescence. J. Plant Breed. Crop Sci..

[167] Chen Y., Li Y., Zhou M., Cai Z., Tambel L.I.M., Zhang X., Chen Y., Chen D. (2019). Nitrogen deficit decreases seed Cry1Ac endotoxin expression in *Bt* transgenic cotton. Plant Physiol. Biochem..

[168] Adamczyk J.J., Sumerford D.V. (2001). Potential factors impacting season-long expression of *Cry1Ac* in 13 commercial varieties of Bollgard cotton. J. Insect Sci..

[169] Adamczyk J.J., Hardee D.D., Adams L.C., Sumerford D.V. (2001). Correlating differences in larval survival and development of bollworms (*Lepidoptera*: *Noctuidae*) and fall armyworms (*Lepidoptera*: *Noctuidae*) to differential expression of Cry1A(c) δ–endotoxin in various plant parts among commercial cultivars of transgenic *Bacillus thuringiensis* cotton. J. Econ. Entomol..

[170] Adamczyk J.J., Perera O., Meredith W.R. (2009). Production of mRNA from the *Cry1Ac* transgene differs among Bollgard lines which correlates to the level of subsequent protein. Transgenic Res..

[171] Zhang L., Shen W., Fang Z., Liu B. (2016). Cry1ab/c in different stages of growth in transgenic rice Bt-shanyou63. Front. Biosci..

[172] Sanahuja G., Banakar R., Twyman R.M., Capell T., Christou P. (2011). *Bacillus thuringiensis*: A century of research, development and commercial applications. Plant Biotechnol. J..

[173] Palma L., Muñoz D., Berry C., Murillo J., Caballero P. (2014). *Bacillus thuringiensis* toxins: An overview of their biocidal activity. Toxins.

[174] Melo A.L., Soccol V.T., Soccol C.R. (2016). *Bacillus thuringiensis*: Mechanism of action, resistance, and new applications: A review. Crit. Rev. Biotechnol..

[175] de Wit P.J.G.M. (1992). Molecular characterization of gene-for-gene systems in plant-fungus interactions and the application of avirulence genes in control of plant pathogens. Annu. Rev. Phytopathol..

[176] Kamoun S., van West P., Vleeshouwers V.G.A.A., de Groot K.E., Govers F. (1998). Resistance of *Nicotiana benthamiana* to *Phytophthora infestans* is mediated by the recognition of the elicitor protein INF1. Plant Cell.

[177] Keller H., Pamboukdjian N., Ponchet M., Poupet A., Delon R., Verrier J.L., Roby D., Ricci P. (1999). Pathogen induced elicitin production in transgenic tobacco generates a hypersensitive response and nonspecific disease resistance. Plant Cell.

[178] Heath M.C. (2000). Nonhost resistance and non-specific plant defenses. Curr. Opin. Plant Biol..

[179] Stalker D.M., Mcbride K.E., Malyj L.D. (1988). Herbicide resistance in transgenic plants expressing a bacterial detoxification gene. Science.

[180] Matten S.R., Reynolds A.H. (2003). Current resistance management requirements for Bt cotton in the United States. J. New Seeds.

[181] Fraser M.J. (2012). Insect transgenesis: Current applications and future prospects. Annu. Rev. Entomol..

[182] Davis M.J., Ying Z. (2004). Development of papaya breeding lines with transgenic resistance to papaya ringspot virus. Plant Dis..

[183] Ilardi V., Nicola-Negri E.D. (2011). Genetically engineered resistance to plum pox virus infection in herbaceous and stone fruit hosts. GM Crop.

[184] Orbegozo J., Solorzano D., Cuellar W.J., Bartolini I., Roman M.L., Ghislain M., Kreuze J. (2016). Marker-free PLRV resistant potato mediated by Cre-loxP excision and RNAi. Transgenic Res..

[185] Chiozza M.V., Burachik M., Miranda P.V. (2020). Compositional analysis of soybean event IND–ØØ41Ø–5. GM Crops Food.

[186] Demaneche S., Sanguin H., Pote J., Navarro E., Bernillon D., Mavingui P., Wildi W., Vogel T.M., Simonet P. (2008). Antibiotic-resistant soil bacteria in transgenic plant fields. Proc. Natl. Acad. Sci. USA.

[187] Song J., Bradeen J.M., Naess S.K., Raasch J.A., Wielgus S.M., Haberlach G.T., Liu J., Kuang H., Austin-Phillips S., Buell C.R. (2003). Gene RB cloned from *Solanum bulbocastanum* confers broad spectrum resistance to potato late blight. Proc. Natl. Acad. Sci. USA..

[188] Yin Z., Hennig J., Szwacka M., Malepszy S. (2004). Tobacco PR-2d promoter is induced in transgenic cucumber in response to biotic and abiotic stimuli. J. Plant Physiol..

[189] Kim Y.S., Lim S., Yoda H., Choi Y.E., Sano H. (2011). Simultaneous activation of salicylate production and fungal resistance in transgenic *Chrysanthemum* producing caffeine. Plant Signal Behav..

[190] Jisha V., Dampanaboina L., Vadassery J., Mithöfer A., Kappara S., Ramanan R. (2015). Overexpression of an AP2/ERF type transcription factor OsEREBP1 confers biotic and abiotic stress tolerance in rice. PLoS ONE.

[191] Coppola M., Corrado G., Coppola V., Cascone P., Martinelli R., Digilio M.C., Pennacchio F., Rao R. (2015). Prosystemin overexpression in tomato enhances resistance to different biotic stresses by activating genes of multiple signaling pathways. Plant Mol. Biol. Rep..

[192] Liu Q., Liu Y., Tang Y., Chen J., Ding W. (2017). Overexpression of NtWRKY50 increases resistance to *Ralstonia solanacearum* and alters salicylic acid and jasmonic acid production in tobacco. Front. Plant Sci..

[193] Klee H.J. (1993). Ripening physiology of fruit from transgenic tomato (*Lycopersicon esculentum*) plants with reduced ethylene synthesis. Plant Physiol..

[194] Taning C.N.T., Arpaia S., Christiaens O., Dietz-Pfeilstetter A., Jones H., Mezzetti B., Sabbadini S., Sorteberg H.G., Sweet J., Ventura V. (2020). RNA-based biocontrol compounds: Current status and perspectives to reach the market. Pest Manag. Sci..

[195] Giudice G., Moffa L., Varotto S., Cardone M.F., Bergamini C., de Lorenzis G., Velasco R., Nerva L., Chitarra W. (2021). Novel and emerging biotechnological crop protection approaches. Plant Biotechnol. J..

[196] Rajam M.V. (2020). RNA silencing technology: A boon for crop improvement. J. Biosci..

[197] Mitter N., Worrall E.A., Robinson K.E., Li P., Jain R.G., Taochy C., Fletcher S.J., Carroll B.J., Lu G.Q., Xu Z.P. (2017). Clay nanosheets for topical delivery of RNAi for sustained protection against plant viruses. Nat. Plants.

[198] Senthil-Kumar M., Mysore K.S. (2011). Caveat of RNAi in plants: The off-target effect. Methods Mol. Biol..

[199] Neumeier J., Meister G. (2021). siRNA Specificity: RNAi mechanisms and strategies to reduce off-target effects. Front. Plant Sci..

[200] Chen J., Peng Y., Zhang H., Wang K., Zhao C., Zhu G., Reddy Palli S., Han Z. (2021). Off-target effects of RNAi correlate with the mismatch rate between dsRNA and non-target mRNA. RNA Biol..

[201] Pandey P., Mysore K.S., Senthil-Kumar M. (2022). Recent advances in plant gene silencing methods. Methods Mol. Biol..

[202] Proctor R.H., Hohn T.M., McCormick S.P. (1995). Reduced virulence of *Gibberella zeae* caused by disruption of a trichothecene toxin biosynthetic gene. Mol. Plant-Microbe Interact..

[203] Daub M.E., Ehrenshaft M. (2000). The photoactivated *Cercospora* toxin cercosporin: Contributions to plant disease and fundamental biology. Annu. Rev. Phytopathol..

[204] Cessna S.G., Sear V.E., Dickman M.B., Low P.S. (2000). Oxalic acid, a pathogenicity factor for *Sclerotinia sclerotiorum*, suppresses the oxidative burst of the host plant. Plant Cell.

[205] Gaj T., Gersbach C.A., Barbas C.F. (2013). ZFN, TALEN, and CRISPR/Cas–based methods for genome engineering. Trends Biotechnol..

[206] Leibowitz M.L., Papathanasiou S., Doerfler P.A., Blaine L.J., Sun L., Yao Y., Zhang C.Z., Weiss M.J., Pellman D. (2021). Chromothripsis as an on-target consequence of CRISPR-Cas9 genome editing. Nat. Genet..

[207] Molla K.A., Sretenovic S., Bansal K.C., Qi Y. (2021). Precise plant genome editing using base editors and prime editors. Nat. Plants.

[208] Nerkar G., Devarumath S., Purankar M., Kumar A., Valarmathi R., Devarumath R., Appunu C. (2022). Advances in crop breeding through precision genome editing. Front. Genet..

[209] Chilcoat D., Liu Z.B., Sander J. (2017). Use of CRISPR/Cas9 for crop improvement in maize and soybean. Prog. Mol. Biol. Transl. Sci..

[210] Sánchez-León S., Gil-Humanes J., Ozuna C.V., Giménez M.J., Sousa C., Voytas D.F., Barro F. (2018). Low-gluten, nontransgenic wheat engineered with CRISPR/Cas9. Plant Biotechnol. J..

[211] Nekrasov V., Staskawicz B., Weigel D., Jones D., Kamoun S. (2013). Targeted mutagenesis in the model plant *Nicotiana enthamiana* using Cas9 RNA–guided endonuclease. Nat. Biotechnol..

[212] Shan Q., Wang Y., Li J., Zhang Y., Chen K., Liang Z., Zhang K., Liu J., Xi J.J., Qiu J.L. (2013). Targeted genome modification of crop plants using a CRISPR/Cas system. Nat. Biotechnol..

[213] van der Oost J., Westra E.R., Jackson R.N., Wiedenheft B. (2014). Unravelling the structural and mechanistic basis of CRISPR–Cas systems. Nat Rev Microbiol..

[214] Lawrenson T., Shorinola O., Stacey N., Li C., Østergaard L., Patron N., Uauy C., Harwood W. (2015). Induction of targeted, heritable mutations in barley and *Brassica oleracea* using RNA–guided Cas9 nuclease. Genome Biol..

[215] Malnoy M., Viola R., Jung M.H., Koo O.J., Kim S., Kim J.S., Velasco R., Nagamangala Kanchiswamy C.D. (2016). DNA-free genetically edited grapevine and apple protoplast using CRISPR/Cas9 ribonucleoproteins. Front. Plant Sci..

[216] Hu X., Wang C., Fu Y., Liu Q., Jiao X., Wang K. (2016). Expanding the range of CRISPR/Cas9 genome editing in rice. Mol. Plant.

[217] Naeem M., Majeed S., Hoque M.Z., Ahmad I.J.C. (2020). Latest developed strategies to minimize the off-target effects in CRISPR-Cas mediated genome editing. Cells.

[218] Honée G. (1999). Engineered resistance against fungal plant pathogens. Eur. J. Plant Pathol..

[219] Zhang H., Zhang J., Wei P., Zhang B., Gou F., Feng Z., Mao Y., Yang L., Zhang H., Xu N. (2014). The CRISPR/Cas9 system produces specific and homozygous targeted gene editing in rice in one generation. Plant Biotechnol. J..

[220] Wang Y., Cheng X., Shan Q., Zhang Y., Liu J., Gao C., Qiu J.L. (2014). Simultaneous editing of three homoeoalleles in hexaploid bread wheat confers heritable resistance to powdery mildew. Nat. Biotechnol..

[221] Zhu J., Song N., Sun S., Yang W., Zhao H., Song W., Lai J. (2016). Efficiency and inheritance of targeted mutagenesis in maize using CRISPR-Cas9. J. Genet. Genom..

[222] Waltz E. (2016). Gene-edited CRISPR mushroom escapes US regulation. Nature.

[223] Li M., Li X., Zhou Z., Wu P., Fang M., Pan X., Lin Q., Luo W., Wu G., Li H. (2016). Reassessment of the four yield–related genes *Gn1a*, *DEP1*, *GS3*, and *IPA1* in rice using a CRISPR/Cas9 system. Front. Plant Sci..

[224] Waltz E. (2016). CRISPR-edited crops free to enter market, skip regulation. Nat. Biotechnol..

[225] Shimatani Z., Kashojiya S., Takayama M., Terada R., Arazoe T., Ishii H., Teramura H., Yamamoto T., Komatsu H., Miura K. (2017). Targeted base editing in rice and tomato using a CRISPR/Cas9 cytidine deaminase fusion. Nat. Biotechnol..

[226] Braatz J., Harloff H.J., Mascher M., Stein N., Himmelbach A., Jung C. (2017). CRISPR-Cas9 targeted mutagenesis leads to simultaneous modification of different homoeologous gene copies in polyploid oilseed rape *(Brassica napus*). Plant Physiol..

[227] Okuzaki A., Ogawa T., Koizuka C., Kaneko K., Inaba M., Imamura J., Koizuka N. (2018). CRISPR/Cas9-mediated genome editing of the fatty acid desaturase 2 gene in *Brassica napus*. Plant Physiol. Biochem..

[228] Langner T., Kamoun S., Belhaj K. (2018). CRISPR crops: Plant genome editing toward disease resistance. Ann. Rev. Phytopathol..

[229] Zhang Z., Ge X., Luo X., Wang P., Fan Q., Hu G., Xiao J., Li F., Wu J. (2018). Simultaneous editing of two copies of *Gh14–3–3d* confers enhanced transgene-clean plant defense against *Verticillium dahliae* in allotetraploid upland cotton. Front. Plant Sci..

[230] Zaidi S.S., Mukhtar M.S., Mansoor S. (2018). Genome editing: Targeting susceptibility genes for plant disease resistance. Trends Biotechnol..

[231] Wang Y., Wang J., Guo S., Tian S., Zhang J., Ren Y., Li M., Gong G., Zhang H., Xu Y. (2021). CRISPR/Cas9-mediated mutagenesis of *ClBG1* decreased seed size and promoted seed germination in watermelon. Hortic. Res..

[232] Hinge V.R., Chavhan R.L., Kale S.P., Suprasanna P., Kadam U.S. (2021). Engineering resistance against viruses in field crops using CRISPR-Cas9. Curr. Genom..

[233] Usman B., Nawaz G., Zhao N., Liao S., Qin B., Liu F., Liu Y., Li R. (2021). Programmed editing of rice (*Oryza sativa* L.) *OsSPL16* gene using CRISPR/Cas9 improves grain yield by modulating the expression of pyruvate enzymes and cell cycle proteins. Int. J. Mol. Sci..

[234] Zhou X., Liao H., Chern M., Yin J., Chen Y., Wang J., Zhu X., Chen Z., Yuan C., Zhao W. (2018). Loss of function of a rice TPR-domain RNA-binding protein confers broad-spectrum disease resistance. Proc. Natl. Acad. Sci. USA.

[235] Oliva R., Ji C., Atienza-Grande G., Huguet-Tapia J.C., Perez-Quintero A., Li T., Eom J.S., Li C., Nguyen H., Liu B. (2019). Broad-spectrum resistance to bacterial blight in rice using genome editing. Nat. Biotechnol..

[236] Jia H., Zhang Y., Orbović V., Xu J., White F.F., Jones J.B., Wang N. (2017). Genome editing of the disease susceptibility gene *CsLOB1* in citrus confers resistance to citrus canker. Plant Biotechnol. J..

[237] Gomez M.A., Lin Z.D., Moll T., Chauhan R.D., Hayden L., Renninger K., Beyene G., Taylor N.J., Carrington J.C., Staskawicz B.J. (2019). Simultaneous CRISPR/Cas9-mediated editing of cassava *eIF4E* isoforms *nCBP-1* and *nCBP-2* reduces cassava brown streak disease symptom severity and incidence. Plant Biotechnol. J..

[238] Chandrasekaran J., Brumin M., Wolf D., Leibman D., Klap C., Pearlsman M., Sherman A., Arazi T., Gal-On A. (2016). Development of broad virus resistance in non-transgenic cucumber using CRISPR/Cas9 technology. Mol. Plant Pathol..

[239] Wang X., Tu M., Wang D., Liu J., Li Y., Li Z., Wang Y., Wang X. (2018). CRISPR/Cas9-mediated efficient targeted mutagenesis in grape in the first generation. Plant Biotechnol. J..

[240] Santillán Martínez M.I., Bracuto V., Koseoglou E., Appiano M., Jacobsen E., Visser R.G.F., Wolters A.A., Bai Y. (2020). CRISPR/Cas9-targeted mutagenesis of the tomato susceptibility gene *PMR4* for resistance against powdery mildew. BMC Plant Biol..

[241] Ortigosa A., Gimenez-Ibanez S., Leonhardt N., Solano R. (2019). Design of a bacterial speck resistant tomato by CRISPR/Cas9-mediated editing of *SlJAZ2*. Plant Biotechnol. J..

[242] Zhang Y., Bai Y., Wu G., Zou S., Chen Y., Gao C., Tang D. (2017). Simultaneous modification of three homoeologs of *TaEDR1* by genome editing enhances powdery mildew resistance in wheat. Plant J..

[243] Khang B. (2016). The regulatory status of genome-edited crops. Plant Biotechnol. J..

[244] Cox D.B.T., Gootenberg J.S., Abudayyeh O.O., Franklin B., Kellner M.J., Joung J., Zhang F. (2017). RNA editing with CRISPR-Cas13. Science.

[245] Aman R., Ali Z., Butt H., Mahas A., Aljedaani F., Khan M.Z., Ding S., Mahfouz M. (2018). RNA virus interference via CRISPR/Cas13a system in plants. Genome Biol..

